# Probiotic Yoghurt Enriched with Mango Peel Powder: Biotransformation of Phenolics and Modulation of Metabolomic Outputs after In Vitro Digestion and Colonic Fermentation

**DOI:** 10.3390/ijms24108560

**Published:** 2023-05-10

**Authors:** Hafza Fasiha Zahid, Akhtar Ali, Alistair R. Legione, Chaminda Senaka Ranadheera, Zhongxiang Fang, Frank R. Dunshea, Said Ajlouni

**Affiliations:** 1 School of Agriculture, Food and Ecosystem Sciences, Faculty of Science, The University of Melbourne, Parkville, VIC 3010, Australia; zahidh@student.unimelb.edu.au (H.F.Z.); akali@student.unimelb.edu.au (A.A.); senaka.ranadheera@unimelb.edu.au (C.S.R.); zhongxiang.fang@unimelb.edu.au (Z.F.); fdunshea@unimelb.edu.au (F.R.D.); 2 Melbourne Veterinary School, Faculty of Science, The University of Melbourne, Parkville, VIC 3010, Australia; legionea@unimelb.edu.au

**Keywords:** mango peel enriched yoghurts, 16s rRNA gene sequencing, colonic fermentation, phenolic catabolism, LC-ESI-QTOF-MS^2^, short-chain fatty acids

## Abstract

This study investigated the health-promoting effects and prebiotic functions of mango peel powder (MPP) both as a plain individual ingredient and when incorporated in yoghurt during simulated digestion and fermentation. The treatments included plain MPP, plain yoghurt (YA), yoghurt fortified with MPP (YB), and yoghurt fortified with MPP and lactic acid bacteria (YC), along with a blank (BL). The identification of polyphenols in the extracts of insoluble digesta and phenolic metabolites after the in vitro colonic fermentation were performed employing LC-ESI-QTOF-MS^2^. These extracts were also subjected to pH, microbial count, production of SCFA, and 16S rRNA analyses. The characterisation of phenolic profiles identified 62 phenolic compounds. Among these compounds, phenolic acids were the major compounds that underwent biotransformation via catabolic pathways such as ring fission, decarboxylation, and dehydroxylation. Changes in pH indicated that YC and MPP reduced the media pH from 6.27 and 6.33 to 4.50 and 4.53, respectively. This decline in pH was associated with significant increases in the LAB counts of these samples. The *Bifidobacteria* counts were 8.11 ± 0.89 and 8.02 ± 1.01 log CFU/g in YC and MPP, respectively, after 72 h of colonic fermentation. Results also showed that the presence of MPP imparted significant variations in the contents and profiles of individual short chain fatty acids (SCFA) with more predominant production of most SCFA in the MPP and YC treatments. The 16s rRNA sequencing data indicated a highly distinctive microbial population associated with YC in terms of relative abundance. These findings suggested MPP as a promising ingredient for utilisation in functional food formulations aiming to enhance gut health.

## 1. Introduction

It has been suggested that consuming more fruits and vegetables is associated with a lower risk of certain chronic diseases such as cancers, metabolic syndrome, cardiovascular diseases (CVD), and other degenerative diseases [1,2]. Thus, diet management has been recommended as a practical approach to decrease the prevalence and progression of these chronic disorders. Nevertheless, explicating the association between nutrition and wellbeing involves a precise insight into the digestive processes through which food products are altered by their interaction with the gut microbiota to exercise biological functioning [3]. Alongside digestion, various enzymatic and chemical reactions, in combination with mechanical agitations, convert food macronutrients (carbohydrates, lipids, and proteins) into absorbable components in the intestinal lumen [3]. Though digestion is a very effective process, some proportion of food components escape the hydrolytic action of enzymes and successive absorption, hence moving to the colonic phase, where the colonic microflora can further interact with them.

Mango peels are a good source of distinct phytochemical compounds including carotenes, phytosterols, and polyphenols [1]. The major complex carbohydrates in mangoes are dietary fiber (DF) and its constituents such as pectin, cellulose, and hemicelluloses, while polyphenols are another key phytochemical substance. These constituents represent the main elements of the non-absorbable fraction of a food matrix. They remain linked to DFs, which are not hydrolysed by human digestive enzymes and is instead transported to the colon where it is subsequently fermented by the gut microbiota [4]. These gut microbes alter complex phenolic substances into low molecular weight components, which exert their advantageous effects through their prebiotic-like actions of modulating the beneficial gut flora such as *Lacticaseibacillus* and *Bifidobacterium* [5]. The gut microbiota will also ferment the DFs to produce short chain fatty acids (SCFA) which exert enhanced beneficial effects on the immune system [6]. During the process of fermentation, colonic bacteria can generate a broad array of compounds that can exert positive or negative influences on gut physiology [7]. Sayago-Ayerdi et al. [8] used a dynamic large intestine fermentation system (TIM-2 model) to demonstrate changes in the gut microbiota induced by mango peels. They reported that the main detected genera were *Bifidobacterium*, *Lacticaseibacillus*, *Dorea*, and *Lactococcus*, and their abundance was dependent on the time of fermentation, whereby *Bifidobacteria* were reported to be highly abundant at 24 h of colonic fermentation.

The present study investigated the stability and faecal bioconversion of polyphenols in plain mango peel powder (MPP) and MPP in yoghurt as a model food system using an in vitro colonic fermentation protocol. Furthermore, the generation of metabolites such as short chain fatty acids (SCFA), the acidification profile, and the corresponding transformation in microbial diversity were analysed.

## 2. Results and Discussions

### 2.1. Identification of Precursor Polyphenols and Phenolic Catabolites in MPP and MPP Fortified Yoghurts before and after In Vitro Colonic Fermentation

In the present work, MPP, YA, YB, and YC sample residues obtained after a simulated in vitro digestion were submitted to in vitro colonic fermentation in the presence of an inoculum prepared from human faeces. The tentative identification of phenolic compounds in the insoluble digesta and phenolic metabolites after in vitro faecal fermentation was monitored at various incubation intervals: 0, 24, 48, and 72 h. Similar to a previous related study [9], no phenolics were detected in the samples of YA except gallic acid. The main sources of phenolic compounds in plain yoghurt (YA) without any added MPP are milk proteins and peptides, usually in very small quantities. Therefore, the sample extracts of YA were not subjected to LC-ESI-QTOF-MS^2^ analyses during the in vitro faecal fermentation stages.

The phenolics detected in experimental blanks (FS + CFM) and present in the samples (MPP, YA, YB, YC) were disregarded, therefore, allowing only those compounds which were particular to the action of the colonic faecal microbiota. The polyphenols detected at time 0h (just after the homogenisation of FS with insoluble digesta) were referred to the fraction of phenolics that remained insoluble during simulated in vitro digestion. However, a wide range of phenolic metabolites were detected during 72 h of fermentation. The LC-ESI-QTOF-MS^2^ used in this study allowed for the identification of 62 phenolic compounds (Figure S1), including phenolic acids, flavonoids, and other polyphenols (Table 1).

#### 2.1.1. Phenolic Acids

Compounds **1**–**8** were detected as hydroxybenzoic acids. The major fragmentation patterns associated with these phenolic acids were [M-CO_2_]^−^ and [M-CO_2_-H_2_O]^−^. Compound **1**, with characteristic parent ions at *m*/*z* 300.9991 and fragment ions at *m*/*z* 257 and *m*/*z* 229, was tentatively identified as ellagic acid [10]. Compounds **2**, **5**, **6**, and **7**, with precursor ions at *m*/*z* 153.0193, 197.0455, 137.0244 and 169.0142, were identified as protocatechuic acid, syringic acid, 4-hydroxybenzoic acid, and gallic acid, respectively, based on authentic standards (Figure S2). Compound **8** exhibited parent ions at *m*/*z* 410.1644 [M-H]^−^ which dissociated into *m*/*z* 105 and *m*/*z* 77, corresponding to 2- hydroxy hippuric acid. Hydroxy hippuric acids are the common compounds associated with the microbial degradation of polyphenols in the colon [11]. A total of seven hydroxycinnamic acids were identified in MPP and yoghurt sample extracts before and after colonic fermentation. Compounds **9**, **11**, and **15** were identified as caffeic acid 4-*O*-glucoronide (*m*/*z* 355.0653), ferulic acid (*m*/*z* 193.0508), and 3-sinapoylquinic acid (*m*/*z* 397.1134), respectively, based on their characteristic molecular weight and fragment ions. These hydroxycinnamic acids were detected only in the samples of MPP (before faecal fermentation) (Table 1).

Compounds **16**, **17**, **18**, and **19** displayed [M-H]^−^ ions at *m*/*z* 181.0505, 193.0869, 209.0828, and 165.0555, and were tentatively identified as, respectively, homovanillic acid, 3-hydroxyphenylvaleric acid, 5-(3′,4′-dihydroxyphenyl)-valeric acid, and 4-hydroxyphenyl-2-propionic acid, based on their MS^2^ fragment ions and molecular weights. These phenolic acids could come from the microbial metabolism of monomeric polyphenols in the gut by following various catabolic pathways such as C-ring fission, dihydroxylation, and oxidation reactions [12,13].

#### 2.1.2. Flavonoids

A total of 16 flavonoids were tentatively identified in the tested samples (Table 1). Compound **26**, a flavone in the samples after the faecal fermentation of MPP, YB, and YC, was tentatively identified as cirsilineol based on its characteristic precursor ions at [M-H]^−^ with *m*/*z* 343.0823 and fragments at *m*/*z* 328 and *m*/*z* 297. This compound has previously been reported to show binding affinities with various cancer biomarkers while stimulating reactive oxygen species apoptosis [14]. Compounds **33** and **34** displayed parent ions at *m*/*z* 255.0663 and *m*/*z* 287.0925, with MS^2^ ions at *m*/*z* 227, 135, and *m*/*z* 119, and were tentatively identified as 2-dehydro-*O*-desmethylangolensin and 5′-Methoxy-*O*-desmethylangolensin, respectively. Setchell et al. [15] demonstrated the production of 2-dehydro-*O*-desmethylangolensin during the in vitro anaerobic faecal incubation of soy isoflavones, particularly daidzein. Compound **37** showed molecular ions at [M-H]^−^ *m*/*z* 301.0717, labelled as hesperetin with MS^2^ ions at *m*/*z* 283 and *m*/*z* 177, and was detected in the colonic fermentation of MPP extracts. Past studies reported that the microbial metabolism of hesperidin and naringenin in lemon peel increased the release of hesperetin during solid-state fermentation [16].

#### 2.1.3. Other Polyphenols

Two compounds [**38** (*m*/*z* 211.0472) and **39** (*m*/*z* 227.0351)] were putatively identified as hydroxycoumarins and corresponded to urolithin B and urolithin A (Table 1). Compound **40** was tentatively identified as *p*-anisaldehyde with *m*/*z* 134.0451 [M-H]^−^ ions that fragmented to produce ions at *m*/*z* 122 and *m*/*z* 109. Compounds **54** to **62** were recognised as lignans and detected before and after the colonic fermentation of MPP, YB, and YC. Lignans are fibre associated polyphenols that act as strong antioxidants.

### 2.2. Bioconversion of Phenolics during Colonic Fermentation of Mango-Based Yoghurts

**Figure 1:**
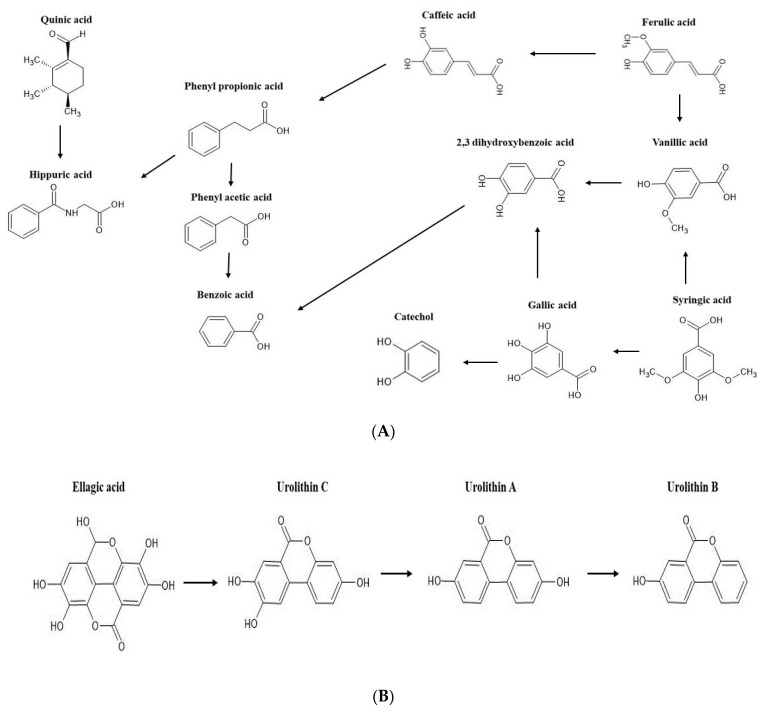

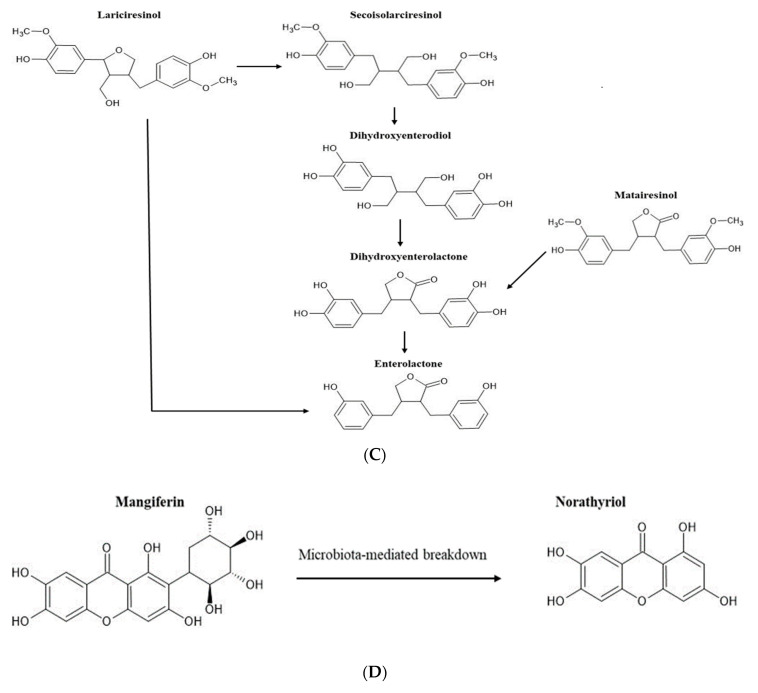
The possible colonic routes of major detected phenolic compounds in mango peel powder (MPP) and respective yoghurt samples. Fecal metabolism of phenolic acids (**A**), catabolism of ellagic acid (**B**), biodegradation of lignans (**C**), and catabolism of mangiferin (**D**).

The mechanisms of interactions between gut flora and dietary polyphenols are characterised by compositional variations in the gut microbiota and/or the production of bioavailable metabolites via the action of gut flora that can modify the potential pharmacological properties of polyphenols. Substantial variations were observed in the phenolic profiles during incubation from 0 to 72 h with faecal matter. For example, ellagic acid was detected only in the insoluble residues of MPP, and protocatechuic acid was found throughout the 72 h of fermentation, whereas some hydroxycinnamic acids such as cinnamic and ferulic acids (*m*/*z* 147.0451 and *m*/*z* 193.0506, respectively) were not detected after 48 h of fermentation. Hernandez-Maldonado et al. [13] reported the rapid fermentation of ferulic, cinnamic, and chlorogenic acids during the colonic fermentation of mango fortified cereal bars. Another study by Dong et al. [17] reported that the methylation and dehydroxylation of syringic acid resulted in the formation of gallic acid during faecal fermentation, which upon further catabolism yielded catechol through decarboxylation and dehydroxylation. Moreover, catechol could also originate from protocatechuic acid, which is a bio-transformation product of gallic acid (compound **7**, *m*/*z* 169.0142) through a dehydroxylation reaction (Figure 1A) [12,18]. Almeida et al. [19] reported that the faecal metabolism of quercetin or its glycosides led to the formation of protocatechuic acid and dihydroxyphenyl acetic acid.

Moreover, the in vitro colonic fermentation of indigestible fractions of MPP, YB, and YC resulted in the appearance of hydroxycoumarins, such as urolithins. As illustrated in Figure 1B, urolithins are the catabolites of ellagic acid. The microbiota mediated conversion of ellagic acid into urolithins is characterised by the hydrolysis of one lactone moiety and the release of water molecule followed by decarboxylation. The generation of urolithins through the degradation of ellagic acid was reported by Mosele et al. [12] and Garcia-Villalba et al. [20] in digested *Arbutus unedo* fruits and pomegranate.

With respect to flavonoids, no detection of flavanols was reported after 24 h of fermentation, but 5-(3′,4′-dihydroxyphenyl)-valeric acid ([M-H]^−^ at *m*/*z* 209.0819) and 3-hydroxyphenylvaleric acid ([M-H]^−^ at *m*/*z* 193.087) derivatives could be produced by dihydroxylation and ring cleavage conversions [21]. Previous studies have shown that catechin and its epimer, epicatechin, are catabolised by intestinal microbiota and converted into dihydroxyphenylpropan-2-ol and dihydroxyphenyl valeric acids, which then undergo dehydroxylation by the action of the colonic microflora, resulting in the generation of (hydroxyl) phenyl propionic acid, phenyl acetic acid, and benzoic acids [18,22].

Lignans, particularly dimethylmatairesinol and lariciresinol, were detected in the residue (solid) fraction of MPP, YB, and YC samples after simulated digestion. In vitro digestion studies suggested that stomach acids and intestinal enzymes do not play a major role in the initial hydrolysis of these lignans, leaving them intact [23]. However, they were biodegraded to enterolignans such as enterodiol and enterolactone in the later stages of faecal fermentation (Figure 1C). Though the precursor compounds have certain physiological effects, their bioconversion into metabolites have considerably greater biological outcomes. The potential anticancer effects of these enterolactones via antioxidant and antiestrogenic activities were proposed in previous studies [24,25]. The release of norathyriol ([M-H]^−^ at *m*/*z* 259.0248) at 48 and 72 h of fermentation indicated the likely breakdown of mangiferin (Figure 1D). According to Li et al. [26] norathyriol inhibits the production of uric acid by targeting organic anion transporters.

### 2.3. Variations in pH and Microbiological Population during Colonic Fermentation

#### 2.3.1. pH

**Figure 2:**
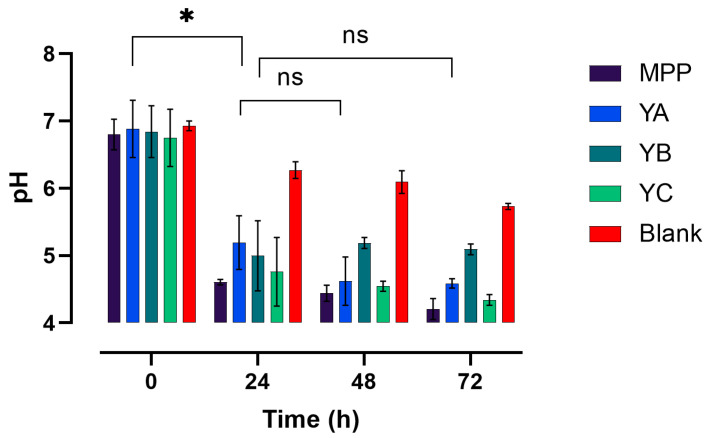
pH variations in the tested samples at various timepoints during colonic fermentation. According to Tukey’s post hoc test, significant difference is indicated by * *p* ≤ 0.05, and ns indicates non-significance. Mango peel powder (MPP), plain yoghurt (YA), mango peel fortified yoghurt (YB), and mango peel fortified probiotic yoghurt (YC).

Changes in pH are important factors that reflect the degree of fermentation. As shown in Figure 2, the initial pH values of the blank, MPP, YA, YB, and YC were about 6.93, 6.83, 6.94, 6.73, and 6.76, respectively, with no significant differences (*p* ≥ 0.05) among them. However, a significant decline (*p* ≤ 0.05) in pH was recorded in the YA, YB, YC, and MPP treatments after the first 24 h of faecal fermentation. The highest decrease (2.19) in pH was observed in MPP samples, while YB, YA, and YC showed a decline of 1.84, 1.69, and 1.99, respectively. A gradual, but less severe, decrease in pH was recorded during the rest of the fermentation in all tested samples, with YB and YC exhibiting the lowest pH of 4.50, followed by MPP, with pH values of 4.53 at 72 h (Figure 2). The lowest acidification (less changes in pH) was detected in the blank (BL) throughout the fermentation period (72 h). These results may suggest that the phenolics and sugars present in MPP and yoghurt were used as sources of carbon for gut microbiota growth and metabolism, which could have triggered the decline in pH. These observations agreed with those reported by Tang et al. [27], who reported a significant decline in the pH of citrus fruits as probiotic fermentation proceeded.

#### 2.3.2. Microbial Population

**Figure 3:**
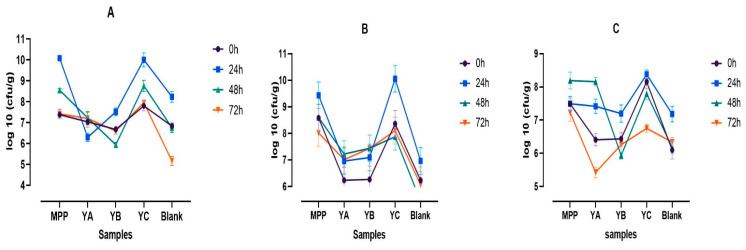
Variations in microbial count (log 10 cfu/g) during the in vitro colonic fermentation of mango peel powder (MPP), plain yoghurt (YA), mango peel fortified yoghurt (YB), and mango peel fortified probiotic yoghurt (YC). (**A**) Lactic acid bacteria, (**B**) Bifidobacteria, and (**C**) Total anaerobes.

The quantitative variations in faecal microbiota during the in vitro colonic fermentation of plain MPP and MPP enriched yoghurt were assessed using the spread plate method. Plate count agar (PCA), MRS agar, and MRS agar enriched with cysteine were applied to examine lactic acid bacteria count (LAB), bifidobacteria, and total anaerobes, respectively (Figure 3A–C). The Bifidobacteria and LAB were selected as they belong to the predominant class of health promoting bacteria and are the producers of SCFA [28]. The recorded microbial abundance elicited by all treatments (YA, YB, YC, and MPP) in comparison with their respective blanks are given in Figure 3. The blank samples [colonic fermentation medium (CFM) + faecal slurry (FS)] showed a significant decline (*p* ≤ 0.05) in the log colony counts of all tested bacteria throughout the fermentation period in comparison with YC and MPP. Similarly, both the YA and YB treatments revealed similar LAB counts (Figure 3A), Bifidobacteria (Figure 3B), and total anaerobic (Figure 3C), but smaller counts than those in YC and MPP at each time of sampling during the 72 h of fermentation.

The initial Bifidobacteria counts in yoghurt enriched with MPP and probiotics (YC) and in plain MPP (Figure 3B) were very close, such as 8.36 and 8.58 log CFU/g, respectively. Furthermore, the Bifidobacteria counts in these two treatments were increased significantly to the maximum counts of 10.06 and 9.44 log CFU/g in YC and plain MPP, respectively, after 24 h of fermentation, followed by a continuous decline. However, the final counts of Bifidobacteria in these treatments remained greater than the counts in YA and TB after 72 h of fermentation (Figure 3B).

Similar patterns of changes in the total anaerobic and LAB counts were detected during the 72 h of fermentation. For example, after the initial increase in the LAB counts in YC at 24 h of fermentation, the counts decreased to 8.95 and 7.88 log CFU/g after 48 h and 72 h of fermentation, respectively (Figure 3A). These results suggested that the presence of mango peel powder in the YC treatments had significant positive prebiotic effects on the growth of LAB during the in vitro fermentation. Such prebiotic effects of MPP were not as significant in yoghurt enriched with MPP only (the YB treatment). This may be due to the fact that prebiotic effects were triggered by the presence of probiotic bacteria in the YC treatment.

The detected positive effect of plain MPP on the population of all tested bacteria could be attributed to the content of phenolic compounds and dietary fibre present in MPP. However, such positive effect was not detected in the YB treatment, where yoghurt was enriched with mango at 2% only. The effect of phenolic compounds on the gut microbiota has been reported by Boto-Ordóñez et al. [29], who suggested that foods rich in phenolic compounds may affect gut microbiota composition and activity by stimulating or inhibiting specific bacterial groups. Another investigation by Gutiérrez-Sarmiento et al. [3] reported that the colonic fermentation of mango-based bars revealed a considerable increase in Firmicutes and Actinobacteria during the initial 24 h with a reduced abundance until 48 h of fermentation.

#### 2.3.3. Changes in Faecal Microbial Diversity in the Presence of MPP

**Figure 4:**
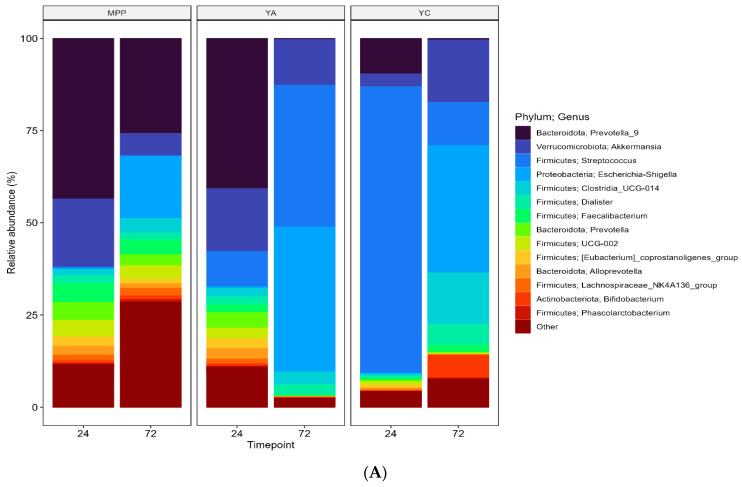

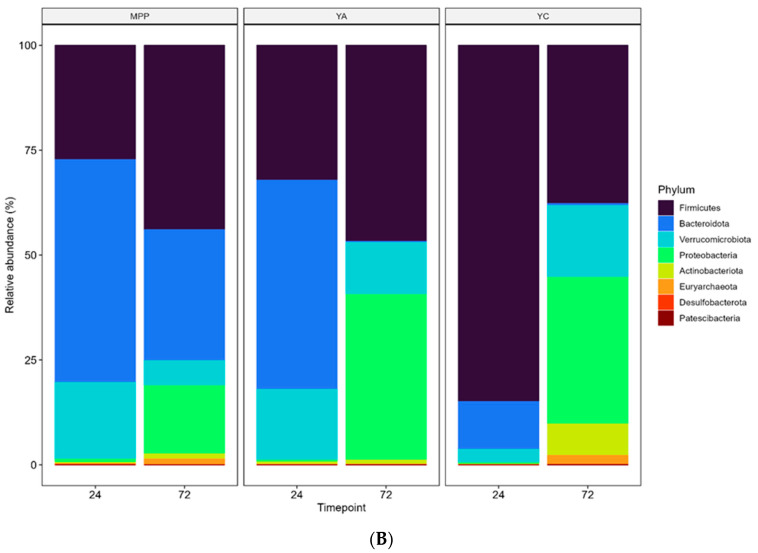
Relative abundance (RA) of genera with those not in the top 14 across the whole population aggregated as ‘Other’ (**A**) and phyla (**B**) present across all fermented samples of mango peel powder (MPP), plain yoghurt (YA), and mango peel fortified probiotic yoghurt (YC) at 24 and 72 h of incubation. Replicates at each timepoint have been merged with the median abundance of each genera displayed.

It has been well established that the human gut microbiota is associated with metabolism, disease development, and immune functions in the body. From the results of the colony count methods and the changes in pH discussed above, it was evident that MPP and YC imparted some positive influences; therefore, fermentation media containing MPP, YC, and YA were assessed for compositional analysis through 16S rRNA sequencing at 24 and 72 h of fermentation. The relative abundance (RA) of Firmicutes, Proteobacteria, Bacteroidetes, Verrucomicrobiota, and Actinobacteria was over 99% at the phyla level (Figure 4B). Higher RA (85.06%) of Firmicutes was measured in YC as compared to YA (32.01%) and MPP (27.10%), particularly at 24 h of colonic fermentation, with Streptococcus being the most abundant genera (Figure 4A). The presence of probiotics and MPP in yoghurt (YC) triggered changes in the abundance and composition of the microbial population and was different to that of YA and MPP. The shift in microbial diversity was most likely promoted by dietary fibre and bound polyphenols that escaped small intestinal digestion. In general, the interactions between phenolics and the gut microbiota during fermentation are demonstrated to generate a series of metabolites, which in turn may contribute to modulating the normal microflora balance [17].

A complete statistical analysis of alpha- and beta-diversity was not possible in this study due to the pilot nature of investigating the effect of the different media on microbiome changes. Therefore, the results of estimated alpha- and beta-diversity are given in the supplementary section of this manuscript (Section S2.3.3, Figures S3 and S4). The primary aim of this test was to stipulate an overview on the role of gut microbiota in the metabolism of polyphenol and probiotic fermented diets. However, in vivo experiments are needed with larger sample sizes to validate the results of the present study.

### 2.4. Short Chain Fatty Acids Production during In Vitro Colonic Fermentation of Yoghurt Enriched with Mango Peel Powder

**Figure 5:**
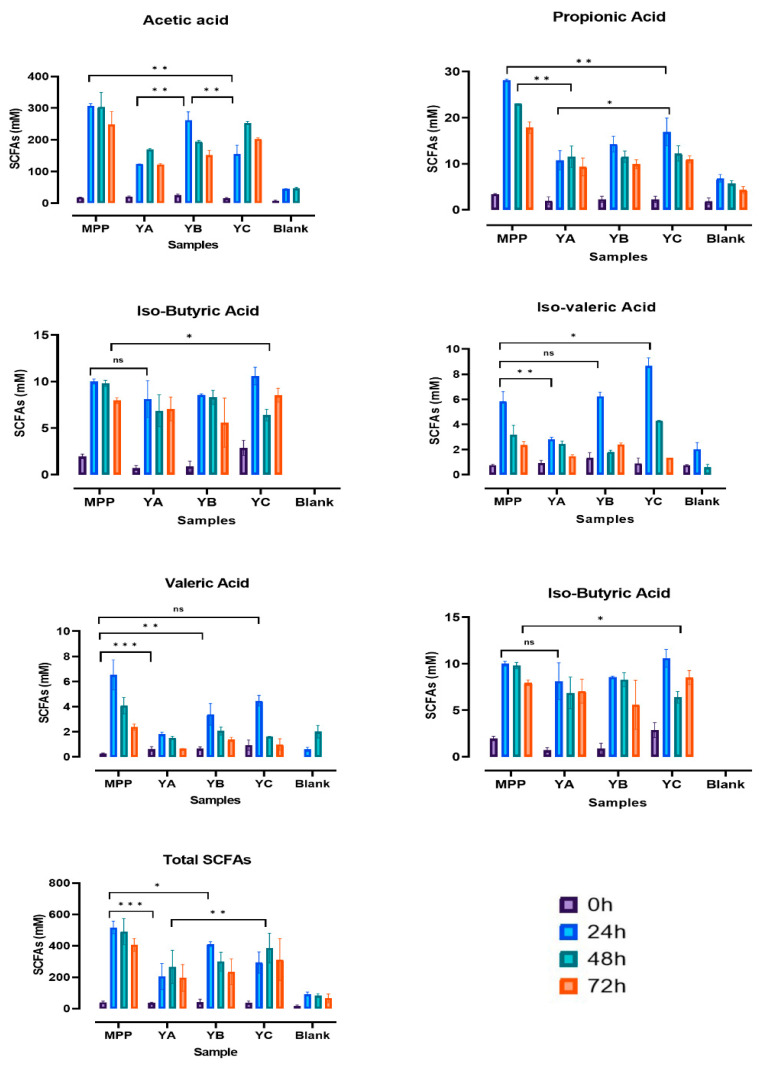
Changes in the concentration of SCFAs (mM) at various incubation timepoints of in vitro colonic fermentation. According to Tukey’s post hoc test, significant differences were indicated by asterisks: * *p* ≤ 0.01, ** *p* ≤ 0.001, *** *p* ≤ 0.0003; ns indicates non-significance. Mango peel powder (MPP), plain yoghurt (YA), mango peel fortified yoghurt (YB), and mango peel fortified probiotic yoghurt (YC).

Short chain fatty acids (SCFAs) are produced by the gut microbiota via the fermentation of compounds that remain undigested when passing through the intestinal tract. The concentration of SCFAs released in the large intestine depends upon the intestinal transit time, composition of the host’s diet, and microbiota [30]. Figure 5 shows the concentrations of SCFAs released during the in vitro colonic fermentation of MPP, YB, YC, and their associated negative control (YA) and blank (FS + CFM).

Data in Figure 5 show that acetic acid was the most prevalent fatty acid in all tested samples, followed by propionic acid and butyric acid. A substantial increase in the levels of acetic acid was observed in MPP (307.31 ± 5.47 mM) and YB (261.25 ± 22.28 mM) after 24 h of fermentation, whereas the production efficacy of AA in YC delivered higher concentrations (252.46 ± 4.43 mM) subjected to 48h of colonic fermentation (Figure 5). On the other hand, significantly (*p* ≤ 0.05) lower concentrations of acetic acid were produced in YA (123.30 ± 0.62 mM) and blank (45.05 ± 0.36 mM) during the first 24 h of incubation, with an increase of 27% in the concentration of acetic acid in YA during the next 24 h (48 h of fermentation). This higher generation of acetic acid in the presence of MPP and YB could be attributed to the fibres present in MPP. This predominant formation of acetic acid is in accordance with the studies of Hernandez-Maldonado et al. [13] and Freire et al. [31] in which mango-based bars and goat milk fortified with grape juice pomace, respectively, were subjected to colonic fermentation. It is also evident from Figure 5 that the concentration of acetic acid was affected by fermentation time and declined significantly (*p* ≤ 0.05) when the fermentation time extended from 48 to 72 h. These results align well with the findings of Granado-Serrano et al. [32] and Hossain et al. [33], where the authors reported maximum generation of SCFAs between 0 and 48 of colonic fermentation. Previous studies indicated increased production of SCFAs in the presence of different probiotic strains, e.g., *B. animalis* subsp. *lactis* GCL2505 was reported to greatly enhance the levels of SCFAs in the gut [34]. In addition, the enhanced release of acetic acid during the colonic fermentation of MPP and MPP fortified yoghurts could also be attributed to mango flavonoids, which were bio-degraded to various small molecule phenolics such as phenyl valeric acids, phenyl propionic acid, and phenyl acetic acids. These findings were substantiated by recent studies on citrus flavanones, indicating the reduced bioavailability of citrus flavanones leading them to surpass the digestive tract to the colon and subsequently undergo hydrolysis by the gut microbiota into phenolic catabolites, consequently enhancing the levels of SCFAs [27,35].

Figure 5 also reveals that the highest concentrations of propionic acid and butyric acid were achieved after 24 h of fermentation, followed by a gradual decline until 72 h in all treatments except MPP, which showed a small but insignificant (*p* ≥ 0.05) increase of 0.67 mM in the levels of BA between 48 and 72 h of fermentation. However, compared to acetic acid, significantly (*p* ≤ 0.05) lower amounts of butyric and propionic acid were produced by all the tested samples. The contents of propionic acid were highest at 24 h of colonic fermentation in all the tested substrates except YA, where it reached a maximum value of 11.53 ± 1.89 mM after 48 h of colonic incubation. The maximum concentration of butyric acid was achieved by MPP (32.79 ± 2.01 mM) during the first 24 h of colonic fermentation, which was 2.2, 2.8, and 3.6 folds higher than its concentrations in YC, YB, and YA, respectively (Figure 5). The enhanced release of these acids from MPP could be possibly due the stimulus provided by the gut microbiota to the phenolics (flavanols) and dietary fibres in MPP [36,37]. Interestingly, synergism between SCFAs and phenolics, for example, BA and gallic acid, has been linked to the modulation of inflammatory signals for treating inflammatory disorders [38]. Analogous to the acetic acid, lower amounts of butyric acid were released in the blank (8.16 ± 0.23 mM) with a gradual decline in production efficacy until 72 h of colonic fermentation. These findings are supported by the study by Herrera-Cazares et al. [37], who reported that the in vitro colonic fermentation of mango bagasse and mango bagasse based functional confections produced increased concentrations of acetate and butyrate.

Regarding the contents of valeric acid, a similar production pattern to that of propionic acid was observed. However, significantly (*p* ≤ 0.05) lower amounts of valeric acid were detected in comparison to propionic acid. The highest amounts of valeric acid (6.52 ± 0.96 mM) were achieved in MPP at 24 h, followed by YC (4.47 ± 0.35 mM), YB (3.38 ± 0.71 mM), YA (1.79 ± 0.14 mM), and blank (0.59 ± 0.15 mM).

Data in Figure 5 also show the production of varying concentrations of isovaleric and isobutyric acids with respect to the different treatments. Unlike MPP, the highest amounts of isovaleric and isobutyric acids were produced by YC at 10.59 ± 0.78 mM and 8.65 ± 0.53 mM, respectively. This could possibly be explained by the fact that varying proportions of certain non-digestible fibres in the substrates may result in various patterns of SCFA production [39]. These results confirmed that amongst the SCFAs formed during the in vitro colonic fermentation, acetic, butyric, and propionic acids were the major SCFAs produced by the colonic microbiota, with higher concentrations, while valeric, isobutyric, and isovaleric acids were the minor SCFAs, in low concentrations. These findings agree with recent results by Loo et al. [40] and Tamargo et al. [41], who investigated in vitro and in vivo colonic fermentation using sugarcane polyphenol and cranberry extracts as substrates. However, Rios-Covian et al. [30] reported that in the hindgut, SCFAs were produced in the order of acetate > propionate > butyrate, which was different from the order of acetate > butyrate > propionate in the present study (Figure 5). This could possibly be attributed to the synergism/antagonism between SCFAs and other phytochemicals and/or microbial cross-feeding in different fermentation systems [37].

## 3. Materials and Methods

### 3.1. Materials

A commercial yoghurt starter culture (YOFLEX^®^—consisting of *Streptococcus thermophilus* and *Lactobacillus delbrueckii* subsp. bulgaricus) and three probiotic strains (*Lacticaseibacillus rhamnosus* LGG, *Lacticaseibacillus casei* 431, *Bifidobacterium animalis* subsp. *lactis* BB-12) were kindly supplied by Chr. Hansen, Bayswater, VIC, Australia. Powdered skim milk (Australian instant skim milk powder, Coles) was obtained from a local supermarket in Melbourne, VIC, Australia. Pancreatin was purchased from Alfa Aesar (Ward Hill, MA, USA). Mangiferin, pyrogallol, gallic acid, caffeic acid, 3-hydroxy phenyl acetic acid, 3-(2-hydroxyphenyl) propionic acid, porcine pepsin, α-amylase from aspergillus oryzae, p-nitrophenyl-α-d-glucopyranoside, bile salts, LC-MS grade formic acid, acetic acid, 4-methyl valeric acid, guar, casein, tryptone, cysteine HCl, peptone, mucin, pectin, potato starch, yeast extract, and MRS agar were acquired from Sigma–Aldrich (Castle Hill, NSW, Australia). Sodium carbonate (anhydrous), HCl, NaHCO_3_, NaOH, (NH_4_)_2_CO_3_, KCl, NaCl, KH_2_PO_4_, MgCl_2_(H_2_O)_6_, CaCl_2_, K_2_HPO_4_, Na_3_PO_4_, MgSO_4_.7H_2_O Tween 80, orthophosphoric acid, acetonitrile, and methanol of HPLC grade were acquired from Chem-supply Pty Ltd. (Melbourne, VIC, Australia).

### 3.2. Methods

#### 3.2.1. Preparation of Probiotic Cultures and Mango Peel Powder

The probiotic strains of *L. rhamnosus* (LGG^®^), *L*. *casei* (431^®^), *B. lactis* (Bb-12^®^), and yoghurt starter culture (SC) (YOFLEX^®^) were activated under anaerobic conditions in de Man Rogosa Sharp (MRS) broth at 37 °C for 48 h, and the cultures were harvested via centrifugation as described by Zahid et al. [42,43]. The mango peels were transformed into powder form using a freeze-drying operation at −48 °C (Dynavac engineering FD3 freeze-drier, Belmont, Australia) and subsequently ground to a uniform particle size of 250 µm [42] using a laboratory coffee grinder (Multigrinder EMO405, Sunbeam, Melbourne, Australia).

#### 3.2.2. Yoghurt Samples Preparation

Yoghurt mixes (stirred type) were prepared in triplicates and labelled yoghurt A (YA), yoghurt B (YB), and yoghurt C (YC) according to the method of Zahid et al. [43]. YA represented the negative control and contained a starter culture (SC) only, YB consisted of SC along with added MPP at a predetermined concentration of 2%, and YC consisted of yoghurt B enriched with each of the tested probiotics at a concentration of 1% (*v*/*v*). Plain MPP was also included in the study as a positive control. All the treatments were prepared in triplicate.

#### 3.2.3. In Vitro Gastrointestinal Digestion and Colonic Fermentation of MPP and MPP Fortified Yoghurts

The simulated fluids for oral, gastric, and small intestinal digestion phases were prepared using a mix of electrolytes (Cl^−^, Na^+^, K^+^, H_2_PO_4_^−^, Mg^2+^, Ca^2+^, NH_4_^+^, and HCO_3_^−^) at variable concentrations. All the samples and controls were subjected to a three-step sequential digestion model using INFOGEST protocol as explained in our previous study [44]. After in vitro digestion, the residue (indigestible fraction) was exposed to colonic fermentation under anaerobic conditions [44].

The procedure of this in vitro colonic fermentation study was approved by the Ethics Advisory Group (ID: 1954660.1) in the Faculty of Science, The University of Melbourne. The colonic fermentation medium (CFM) was made of guar (1.0 g), bile salts (0.4 g), casein (3 g), CaCl_2_ (0.11 g), KCl (4.5 g), KH_2_PO_4_ (0.5 g), K_2_HPO_4_ (0.5 g), cysteine HCl (0.8 g), mucin (4 g), MgSO_4_.7H_2_O (1.23 g), NaCl (4.5 g), NaHCO_3_ (1.5 g), pectin (2 g), peptone (5 g), potato starch (5 g), tryptone (5 g), yeast extract (4.5 g), and Tween 80 (1.0 mL) dissolved in Milli-Q water, and the volume was made up to 1000 mL. The medium was adjusted to pH 6.9 ± 1.0 and autoclaved at 121 °C for 20 min.

Two healthy donors provided freshly defecated faeces. The donors specified that they had not used probiotics or antibiotics in the prior three months and were free of gastrointestinal conditions at the time of sample collection. The faeces were transferred to the lab on ice, combined, and stomached with 0.1 M sterilised phosphate buffer (pH 7.0) in a stomacher mixer (Bagmixer 400, Interscience, Saint-Nom, France). The obtained mixture was then sifted using a cheesecloth to develop one faecal slurry at a proportion of 20:80 (*w*/*w*) faeces:buffer. In 50 mL N_2_ flushed tubes, 5 mL aliquots of faecal slurry were distributed in each tube along with 0.5 g of the intestinal residue (insoluble intestinal fraction) and mixed with 5 mL of the prepared CFM. The tubes were firmly sealed and put in an anaerobic shaking incubator (ZWTR-240, Labwit, China) for 72 h at 120 rpm, 37 °C in the absence of oxygen (anaerobiosis). The anaerobic environment was created in anaerobic jars (BD BBL^™^ Gas Pak^™^, Mississauga, Ontario, Canada) through an anaerobic gas generator (AN 0010W, Oxoid^®^). A blank test was carried out by inoculating a mix of CFM and FS at 1:1 ratio, without MPP or yoghurt samples to correct the contribution of reagents. The sample tubes were taken and analysed at various intervals (0, 24, 48, and 72 h) during fermentation. The tubes were immediately placed in an ice bath to stop the fermentation process. Each sample was run in quadruplicate for every condition tested. The supernatant fraction from each tube was obtained following centrifugation (10,000× *g*, 15 min, 4 °C) and stored at −80 °C until further analyses of phenolic metabolites, microbial count and composition, generation of short chain fatty acids, and pH variations.

#### 3.2.4. Analysis of Phenolic Metabolites

##### Extraction of Phenolics

The extraction of polyphenols from faecal digesta was adapted from Zahid et al. [44] with slight changes. Briefly, 0.5 mL of faecal digesta was mixed with acidified methanol/water (8:20 acidified with 0.01% conc. HCl). The mixtures were vortexed (Ultra Turax T25 D S5, IKA, Germany) and incubated overnight under shaking conditions at 120 rpm, 4 °C. The separation of supernatants was achieved by centrifugation for 15 min at 5000× *g*, 4 °C.

##### Qualitative Analysis of Polyphenols Using LC-ESI-QTOF-MS^2^

The extracted phenolic fractions were characterized by using an Agilent 6520 Accurate-Mass QTOF interfaced with an ionisation (ESI) source and provided with Agilent HPLC 1200 series (Agilent, Santa Clara, CA, USA). HPLC separation was performed on a Synergi Hydro-RP (4 μm, 4.6 mm, 250 mm) column (Phenomenex, Lane Cove, NSW, Australia) with a pore size of 80 Å. The mobile phases used were water (Buffer A) and acetonitrile (Buffer B) acidified with 0.1% formic acid. The gradient elution programme was set at B: 0–10 min, 10–20%; 10–20 min, 20–25%; 20–30 min, 25–30%; 30–40 min, 30–45%; 40–50 min, 45–60%; 50–60 min, 60–80%; 60–65 min, 60–80%; 65–67 min, 90–100%; 67–70 min, 100–10%. The sample volume was set at 10 µL with an elution flow rate of 0.6 mL/min. Accurate-Mass QTOF 6520 was set to function in a negative ionisation mode (ESI) at a capillary voltage of 3500 V with a scanning speed of 250 spectra/s, and the mass spectra were attained through full scan within the mass range of 100–1000 *m*/*z* in MS/MS mode. Nitrogen (N_2_) was used as a nebuliser and drying gas at a temperature of 325 °C with a flow rate of 9 L/min, 10, 20, and 40 eV collision energies and a nebuliser pressure of 45 psi [9] to achieve the fragmentation of metabolites. The identification of the phenolic metabolites was carried out with the help of the Agilent MassHunter Workstation Quality Analysis Software (version B.06.00), Personal Compounds Database and Library for metabolites (PCDL), PubChem (https://pubchem.ncbi.nlm.nih.gov/), accessed on 10 July 2022 and FooDB (https://foodb.ca/) accessed on 11 July 2022 [45].

##### 3.2.5. pH and Microbiological Analysis

Samples collected at various time points during colonic fermentation (0 h, 24 h, 48 h and 72 h) were subjected to pH monitoring using a pH meter (HI5221, Hanna, Woonsocket, RI, USA). The variations in faecal bacteria composition were assessed by analysing total aerobic count, lactic acid bacteria (LAB), and total anaerobic count using plate count agar (PCA), MRS agar, and MRS agar enriched with cysteine, respectively [44,46]. The initial bacterial count (blank) was carried out using a mixture of faecal slurry (FS) and sterile basal medium (CFM) prepared at 1:1 ratio, (*v*/*v*). All samples were serially diluted using 0.1% sterile peptone water, spread plated, and incubated (aerobically and aerobically) at 37 °C for 48 h.

##### 3.2.6. 16S rRNA Sequencing Analysis

A subset of samples was used for preliminary investigation into microbial diversity in different sample sets. Two replicates each from MPP, YA, and YC at 24 h and 72 h were used for this purpose. DNA was extracted from 200 µL of the selected fermented samples using the DNeasy PowerSoil Pro Kit (QIAGEN, Venlo, The Netherlands) as per the manufacturer’s instructions. Extracted DNA was subsequently processed through a Zymo DNA Clean and Concentrate kit (Zymo Research, Irvine, CA, USA) as the per manufacturer’s instructions to obtain suitable quality DNA for amplicon sequencing. The V3–V4 hypervariable region of the 16S rRNA gene was amplified and sequenced using primers 341F (5′-CCT AYG GGR BGC ASC AG-3′) and 806R (5′-GGA CTA CNN GGG TAT CTA AT-3′) [47] at the Australian Genome Research Facility (AGRF, Melbourne, Australia). Amplicons were barcoded, pooled, and paired end (2 × 300 bp) sequenced on the Illumina MiSeq platform using the Nextera XT Indexes (Illumina, San Diego, CA, USA).

Raw data were demultiplexed and converted into FASTQ format by the Illumina conversion software (version v2.2.68) at AGRF. The Quantitative Insights into Microbial Ecology 2 (QIIME2) v2021.2.0 software was used for downstream processing [48]. Amplicon sequence variants (ASVs) were generated using DADA2 [49]. In DADA2, the demultiplexed data were quality filtered, primer trimmed, denoised, and processed to remove chimeras. Through DADA2, forward and reverse reads were truncated to 260 and 220 bp, respectively, to maximise the average read numbers retained after processing.

Taxonomic classification on the resulting ASVs was performed using a naïve bayes classifier within QIIME2 [50] using the SILVA 138.1 [51] 16S rRNA database clustered to 99% similarity and trimmed to the V3-V4 region using the primer sequence outlined above. Data were imported into the phyloseq package in R [52], and alpha and beta diversity metrics were investigated, as was the relative abundance of taxa between different groups. Alpha diversity was estimated from counts using the ‘estimate richness’ function in phyloseq, whilst beta-diversity was investigated using data subsampled to 43,338 reads (the minimum across the sample set at which all other samples had reached a plateau in respect to observed ASVs). Beta-diversity analysis utilised UniFrac ordination [52] for principal coordinate analysis.

#### 3.2.7. Determination of SCFA

Evaluation of the SCFA concentrations in colonic digesta was performed adopting the procedure of Loo et al. [40] with minor changes. After colonic fermentation, supernatants (1.5 mL) collected after centrifugation were combined with four volumes of an internal standard mixture containing 1.59 mmol/L of 4-methyl valeric acid mixed with formic acid and orthophosphoric acid (1% both) and vortexed for 30 s. From the final mixture, 1 mL of each sample was dispensed into 1.5 mL flip capped tubes and centrifuged (10,000× *g*, 10 min at 4 °C). The supernatants were separated and stored at 4 °C until analysis. Acetic, butyric, propionic, iso-valeric, valeric, iso-butyric, and heptanoic acids were used as analytical standards to create the standard curves [44].

Aliquots (2 μL) of the sample and standards were injected into a gas chromatograph (7890B Agilent, CA, USA) that was fitted with a capillary column of 12 × 0.53 mm internal diameter (ID) and a film thickness of 0.5 µm (SGE BP21, SGE International, Ringwood, VIC, Australia, P/N 054473), a flame ionisation detector (FID), an autosampler (Gilson GX-271, Gilson Inc., Middleton, WI, USA), and an autoinjector. The FID and injection port were set at temperatures of 240 and 200 °C, respectively. Helium was applied as a carrier gas at a flow rate of 14.4 mL/min along with hydrogen, nitrogen, and air as makeup gases.

#### 3.2.8. Statistical Analysis

All collected data apart from microbiome data were analysed using the GraphPad Prism statistical package version 9.5.0 (GraphPad Software LLC, Inc., San Diego, CA, USA). Two-way analysis of variance (ANOVA) was performed, and the difference between means was determined using Tukey’s post hoc test at 95% confidence interval. The quantitative measurements were performed in triplicate with at least two measurements for each sample, and the results were expressed as mean ± standard deviation. The qualitative data analysis was performed as replicates with a single measurement within each sample.

## 4. Conclusions

The results of the present work demonstrated that phytochemicals in mango peel powder have prebiotic effects during colonic fermentation. Insoluble fractions of the digesta were susceptible to further biotransformation during the in vitro colonic fermentation with human faecal microbiota. The catabolism of polyphenols resulted in the production of several phenolic metabolites such as hippuric acid, hydroxyphenyl acetic acid, catechol, protocatechuic acid, urolithins, enterolactones, and norathyriol. The prebiotic-like functions of mango peels were reflected by a significant increment in the lactic acid bacterial counts. Nevertheless, these actions were highly dependent upon the structural composition of the food matrices and the incubation times. These findings were further supported by the reduction in the pH of fermentation media and the generation of SCFAs, where acetic, propionic, and butyric acids were the major SCFAs produced. Additionally, the 16S rRNA analyses of the microbiota after the in vitro colonic fermentation of plain MPP and yoghurt enriched with MPP revealed some modifications in various bacterial groups. These findings presented insights into the significant roles of MPP as prebiotics when used as a plain ingredient or in dairy food systems after human consumption. However, in vivo studies are needed in the future to gain a deep understanding of the microbiota mediated gut well-being of MPP in human diet.

## Figures and Tables

**Table 1:** LC-ESI-QTOF-MS^2^ identification of phenolic compounds and metabolites in MPP and MPP fortified yoghurt samples before and after in vitro colonic fermentation at different incubation timepoints (0 h, 24 h, 48 h, and 72 h).

Phenolic Classes	Proposed Compounds	Molecular Formula	RT (min)	Molecular Weight	Theoretical (*m*/*z*)	Observed (*m*/*z*)	Mass Error (ppm)	MS/MS Product Ions	Samples
**Phenolic acids**									
**Hydroxybenzoic acids**									
1	4-Hydroxybenzoic acid	C_7_H_6_O_3_	4.295	138.0317	137.0244	137.0247	2.1894	93	MPP-C1
2	Gallic acid	C_7_H_6_O_5_	7.882	170.0215	169.0142	169.0135	−4.1417	125	MPP-I, YB-I, YC-I, MPP-C1 *, MPP-C2 *, YC-C1, YC-C2, YB-C1, YB-C2
3	Protocatechuic acid	C_7_H_6_O_4_	12.235	154.0266	153.0193	153.0195	1.307	109	MPP-I, MPP-C1, MPP-C2, MPP-C3 *
4	Gallic acid 3-O-gallate	C_14_H_10_O_9_	14.365	322.0325	321.0252	321.0258	1.869	303, 275, 169	MPP-I, YB-I, YC-I
5	3-O-Methylgallic acid	C_8_H_8_O_5_	15.105	184.0372	183.0299	183.0302	1.6391	123	MPP-I, YB-I, YC-I
6	Ellagic acid	C_14_H_6_O_8_	20.059	302.0063	300.9991	300.9976	−4.6512	257, 229	MPP-I
7	Syringic acid	C_9_H_10_O_5_	20.168	198.0528	197.0455	197.0465	5.075	182, 153	MPP-I, MPP-C1, MPP-C2, MPP-C3
8	2-Hydroxyhippuric acid	C_9_H_9_NO_4_	48.326	411.1717	410.1644	410.1615	−7.0703	105, 77	MPP-C1, YC-C1, YC-C2
**Hydroxycinnamic acids**									
9	3-Feruloylquinic acid	C_17_H_20_O_9_	4.104	368.1107	367.1034	367.1032	−0.5448	298, 288, 192, 191	MPP-C1, MPP-C2, MPP-C3, YC-C1, YC-C2
10	3-Sinapoylquinic acid	C_18_H_22_O_10_	4.104	398.1213	397.114	397.1134	−1.5109	379, 351, 223	MPP-C3
11	Caffeoyl tartaric acid	C_13_H_12_O_9_	4.107	312.0481	311.0408	311.041	0.643	267, 247, 179	YB-C1, YB-C2
12	Ferulic acid	C_10_H_10_O_4_	4.124	194.0579	193.0506	193.0508	1.036	178, 149, 134	MPP-I, MPP-C2, MPP-C3
13	Caffeic acid 4-*O*-glucuronide	C_15_H_16_O_10_	9.134	356.0744	355.0671	355.0653	−5.0695	179	MPP-I, MPP-C1, MPP-C3
14	1,2-Disinapoylgentiobiose	C_34_H_42_O_19_	10.786	754.232	753.2247	753.228	4.3812	531, 369, 207, 175	YB-I, YC-I, YC-C1, YB-C1
15	p-Coumaric acid	C_9_H_8_O_3_	12.814	164.0473	163.04	163.0401	0.6133	195, 177, 145, 117	MPP-I, YB-I, YB-C1, YB-C2, MPP-C1
**Hydroxyphenyl acetic acids**									
16	Homovanillic acid	C_9_H_10_O_4_	6.081	182.0579	181.0506	181.0505	−0.5523	137, 122	MPP-C1, MPP-C2, MPP-C3, YC-C1, YC-C2, YB-C1, YB-C2
**Hydroxyphenylpentanoic acids**									
17	5-(3′,4′-dihydroxyphenyl)-valeric acid	C_11_H_14_O_4_	20.71	210.0892	209.0819	209.0828	4.3045	191, 165, 135	MPP-C1, MPP-C2, MPP-C3, YC-C1, YC-C3, YB-C1, YB-C3
18	3-Hydroxyphenylvaleric acid	C_11_H_14_O_3_	30.542	194.0943	193.087	193.0869	−0.5179	175, 149, 59	MPP-C1, MPP-C2, MPP-C3, YC-C1, YC-C3, YB-C1, YB-C3
**Hydroxyphenylpropanoic acids**									
19	Dihydroferulic acid 4-sulfate	C_10_H_12_O_7_S	4.812	276.0304	275.0231	275.0239	2.9088	206	MPP-I
20	Dihydroferuloylglycine	C_12_H_15_NO_5_	13.673	253.095	252.0877	252.0886	3.5702	149, 100	YB-I, YC-I
21	4-Hydroxyphenyl-2-propionic acid	C_9_H_10_O_3_	19.76	166.063	165.0557	165.0555	−1.2117	121, 119, 93	MPP-C1, MPP-C2, MP-C3, YC-C1, YB-C1
**Flavonoids**									
**Flavanols**									
22	(-)-Epigallocatechin 3′-O-glucuronide	C_21_H_22_O_13_	4.16	482.106	481.0987	481.0979	−1.6629	149, 121	MPP-I, MPP-C1
23	(-)-Epigallocatechin	C_15_H_14_O_7_	4.17	306.074	305.0667	305.0678	3.6058	261, 219	MPP-C1
24	4′-O-Methylepigallocatechin	C_16_H_16_O_7_	4.206	320.0896	319.0823	319.0799	−7.5216	181, 137, 125	MPP-I, YB-I, YC-I
**Flavones**									
25	7,4′-Dihydroxyflavone	C_15_H_10_O_4_	3.811	254.0579	253.0506	253.0516	3.9518	211, 135, 119	MPP-C1
26	6-Hydroxyflavone	C_15_H_10_O_3_	4.11	238.063	237.0557	237.0559	0.8437	208, 193	MPP-C1, YC-C1
27	Cirsilineol	C_18_H_16_O_7_	19.171	344.0896	343.0823	343.0824	0.2915	328, 297	MPP-C1, YC-C1, YC-C2, YC-C3, YB-C1
28	3,4′,7-Tetrahydroxyflavone	C_15_H_10_O_6_	36.661	286.0477	285.0404	285.0399	−1.7541	287, 209	MPP-I
**Flavonols**									
29	Kaempferide	C_16_H_11_O_6_	4.809	299.0556	298.0483	298.0491	2.6841	284, 255, 163, 107	MPP-C1, MPP-C2
30	Kaempferol 3-O-rhamnoside	C_21_H_19_O_10_	5.637	431.0978	430.0905	430.0906	0.2325	285	YB-C3, YC-C3
31	Quercetin	C_15_H_10_O_7_	31.9	302.0426	301.0353	301.034	−4.3184	127, 285	MPP-I, YC-I
32	Isorhamnetin	C_16_H_12_O_7_	39.883	316.0583	315.051	315.052	3.1741	300, 151, 107	MPP-C1
**Isoflavonoids**									
33	5′-Methoxy-O-desmethylangolensin	C_16_H_16_O_5_	8.738	288.0998	287.0925	287.0918	−2.4382	119	MPP-I, MPP-C1
34	Violanone	C_17_H_16_O_6_	20.207	316.0947	315.0874	315.0866	−2.539	285, 135	MPP-C2, MPP-C3
35	3′-Hydroxymelanettin	C_16_H_12_O_6_	36.548	300.0634	299.0561	299.0547	−4.6814	284	MPP-I
36	Hesperetin	C_16_H_14_O_6_	36.583	302.079	301.0717	301.0725	2.6572	283, 177	MPP-C1, MPP-C2
37	2-Dehydro-O-desmethylangolensin	C_15_H_12_O_4_	43.652	256.0736	255.0663	255.0642	−8.2332	227, 135	MPP-C1
**Other polyphenols**									
**Hydroxycoumarins**									
38	Urolithin A	C_13_H_8_O_4_	30.788	228.0423	227.0351	227.0349	−0.4405	183	MPP-C1, YC-C1, YC-C2
39	Urolithin B	C_13_H_8_O_3_	38.902	212.0473	211.0472	211.0386	−6.6338	215, 198, 187, 169	YC-C1, YB-C1, YB-C2
**Hydroxybenzaldehydes**									
40	p-Anisaldehyde	C_8_H_8_O_2_	12.814	136.0524	135.0451	135.0449	−1.481	122, 109, 94	MPP-I, YB-I, YC-I
**Hydroxybenzoketones**									
41	Norathyriol	C_13_H_8_O_6_	22.799	260.0321	259.0248	259.0235	−5.0188	241, 231, 189, 109	MPP-I, YB-I, YC-I, MPP-C1, MPP-C2, MPP-C3, YC-C1, YC-C2
**Alkylphenols**									
42	3-Methylcatechol	C_7_H_8_O_2_	12.654	124.0524	123.0451	123.0457	4.8763	281, 187, 165	MPP-C1
43	4-Vinylphenol	C_8_H_8_O	21.237	120.0575	119.0502	119.0501	−0.84	93, 75, 65	MPP-C1, MPP-C2, YC-C1, YC-C3
**Phenolic terpenes**									
44	Rosmadial	C_20_H_24_O_5_	4.719	344.1624	343.1551	343.1545	−1.7485	327, 297	YC-C1, YB-C1
45	Carvacrol	C_10_H_14_O	57.007	150.1045	149.0972	149.0972	0	132, 108	YB-C1
**Cyslitol**									
46	Quinic Acid	C_7_H_12_O_6_	3.991	192.0634	191.0561	191.0559	−1.0468	173, 127, 85	MPP-C3, YC-C2, YC-C3
**Tyrosols**									
47	Hydroxytyrosol	C_8_H_10_O_3_	14.391	154.063	153.0557	153.0544	−8.4936	135, 123	MPP-C1
**Xanthones**									
48	Mangiferin	C_19_H_18_O_11_	13.992	422.0849	421.0776	421.0797	4.9872	331, 301, 259	MPP-I, YB-I, YC-I
49	Mangiferin 6′-gallate	C_26_H_22_O_15_	16.163	574.0959	573.0886	573.0898	2.0939	421	MPP-I, YB-I, YC-I
**Other polyphenols**									
50	Coumestrol	C_15_H_8_O_5_	7.895	268.0372	267.0299	267.0296	−1.1235	266, 211	MPP-C2, MPP-C3
51	Phlorin	C_12_H_16_O_8_	8.923	288.0845	287.0772	287.0778	2.09	272, 237, 179	MPP-I, MPP-C1, MPP-C2
52	Pyrogallol	C_6_H_6_O_3_	10.231	126.0317	125.0244	125.0242	−1.5997	97, 81	MPP-I, YB-I, YC-I, MPP-C1, MPP-C2, YB-C1
**Stilbene**									
53	4-Hydroxy-3,5,4′-trimethoxystilbene	C_17_H_18_O_4_	25.451	286.1205	285.1132	285.1135	1.0522	269, 253, 227	MPP-C1, MPP-C2, MPP-C3, YB-C1, YC-C1, YC-C2
**Lignans**									
54	Lariciresinol	C_20_H_24_O_6_	4.163	360.1573	359.15	359.1494	−1.6706	329	MPP-I, YC-I, YC-C1
55	Arctigenin	C_21_H_24_O_6_	4.444	372.1573	371.15	371.1473	−7.2747	356, 312, 295	MPP-I, YC-C1
56	Schisandrin B	C_23_H_28_O_6_	10.646	400.1886	399.1813	399.1818	1.2526	385, 370, 330, 300	MPP-I, YB-I, YC-I, YB-C1, YC-C1
57	Secoisolariciresinol-sesquilignan	C_30_H_38_O_10_	15.697	558.2465	557.2392	557.2407	2.6918	539, 513, 361	MPP-C1, MPP-C2, MPP-C3
58	Enterodiol	C_18_H_22_O_4_	20.149	302.1518	301.1445	301.1435	−3.3207	253	MPP-I, YC-I
59	Dimethylmatairesinol	C_22_H_26_O_6_	23.989	386.1729	385.1656	385.167	3.6348	372, 369, 357, 329	YB-I, YC-I, YC-C1, YC-C2, YB-C1, YB-C2
60	Enterolactone	C_18_H_18_O_4_	36.442	298.1205	297.1132	297.1146	4.712	279, 131	MPP-I, YB-I, YCI, MPP-C2, MPP-C3, YC-C1, YC-C3, YB-C1, YB-C2
61	7-Hydroxysecoisolariciresinol	C_22_H_30_O_5_	40.342	374.2093	373.202	373.2028	2.1436	357, 327	MPP-C2, YB-C2
62	Schisantherin A	C_30_H_32_O_9_	45.172	536.2046	535.1973	535.1999	4.858	519, 489, 415, 121	YB-C1, YB-C2

MPP-I, YB-I, and YC-I represent the intestinal non digestible fractions of mango peel powder (MPP), mango peel fortified yoghurt (YB) and mango peel fortified probiotic yoghurt (YC), respectively. Whereas MPP-C, YB-C, and YC-C refer to the samples after colonic fermentation of MPP, YB and YC, respectively. * Numbering 1, 2, 3 indicates various incubation time intervals (24 h, 48 h and 72 h, respectively).

## Data Availability

The data are available in the Supplementary Materials.

## Supplementary Materials

The following supporting information can be downloaded at: https://www.mdpi.com/article/10.3390/ijms24108560/s1.
