# Supplementary material for: Probiotic Yoghurt Enriched with Mango Peel Powder: Biotransformation of Phenolics and Modulation of Metabolomic Outputs after In Vitro Digestion and Colonic Fermentation
Source: Int J Mol Sci. 2023 May 10;24(10):8560. doi: 10.3390/ijms24108560 (PMC10218215; doi:10.3390/ijms24108560)
Supplement: Supplementary file 1 [file ijms-24-08560-s001.zip › ijms-2392660-supplementary materials.pdf]

Supplementary Materials

Probiotic yoghurt enriched with mango peel powder: biotransformation of phenolics and modulation of metabolomic outputs after *in vitro* digestion and colonic fermentation

Hafza Fasiha Zahid[1], Akhtar Ali[1], Alistair R. Legione[2], Chaminda Senaka Ranadheera[1], Zhongxiang Fang[1], Frank R. Dunshea[1], and Said Ajlouni[1*]

[1] School of Agriculture, Food and Ecosystem Sciences, Faculty of Science, The University of Melbourne, Parkville, VIC, Australia

[2] Melbourne Veterinary School, Faculty of Science, The University of Melbourne, Parkville, VIC, Australia

*Corresponding author: said@unimelb.edu.au

Supplementary Materials

(a)

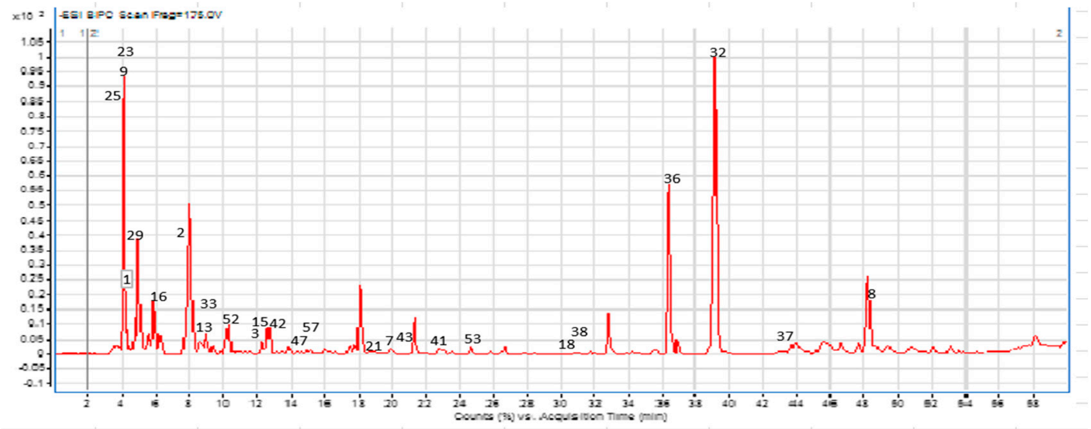

(b)

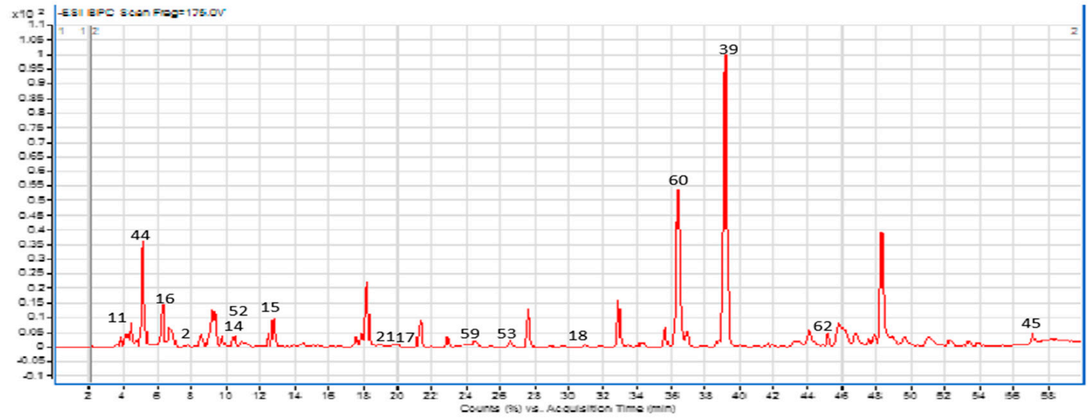

(c)

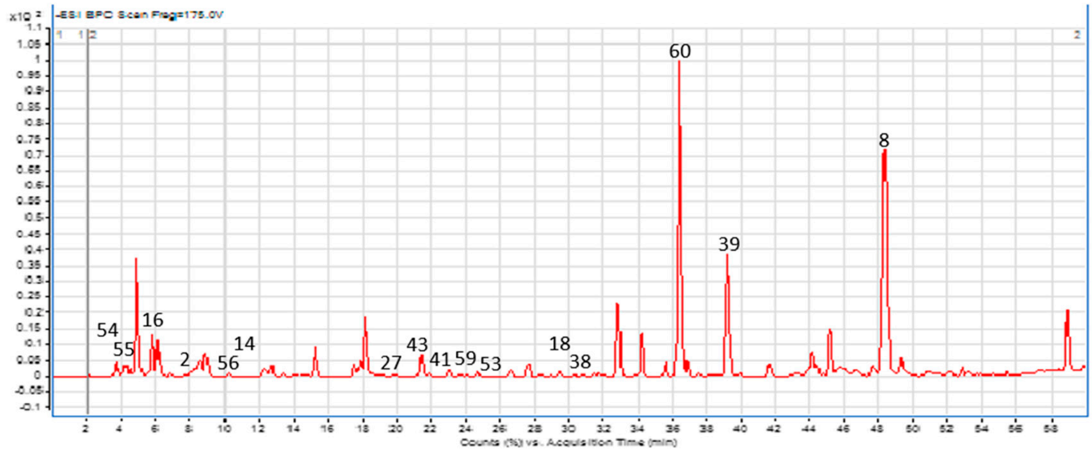

Figure S1. (a) Base peak chromatograms of mango peel powder (MPP), (b) yoghurt fortified with MPP (YB), and (c) yoghurt fortified with MPP and probiotics (YC) after 24h of colonic fermentation. The label numbers refer to the phenolic metabolites present in Table 1.

(a)

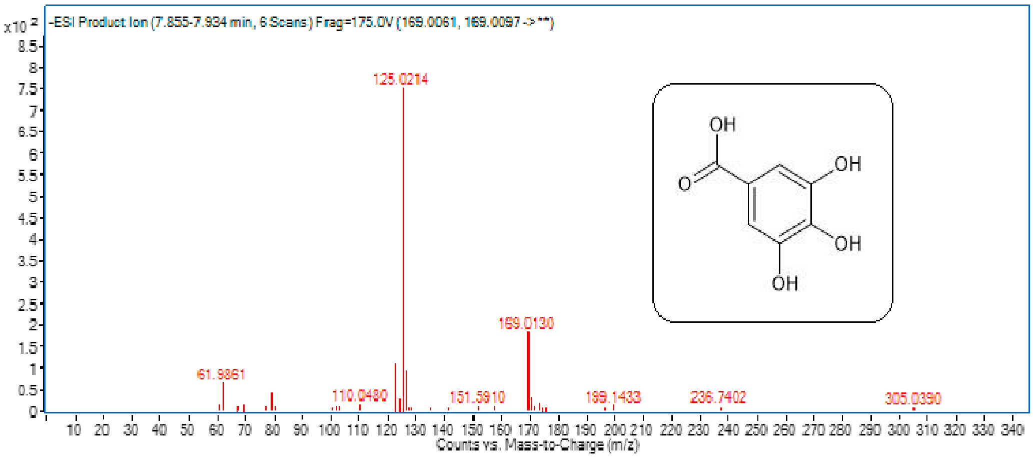

(b)

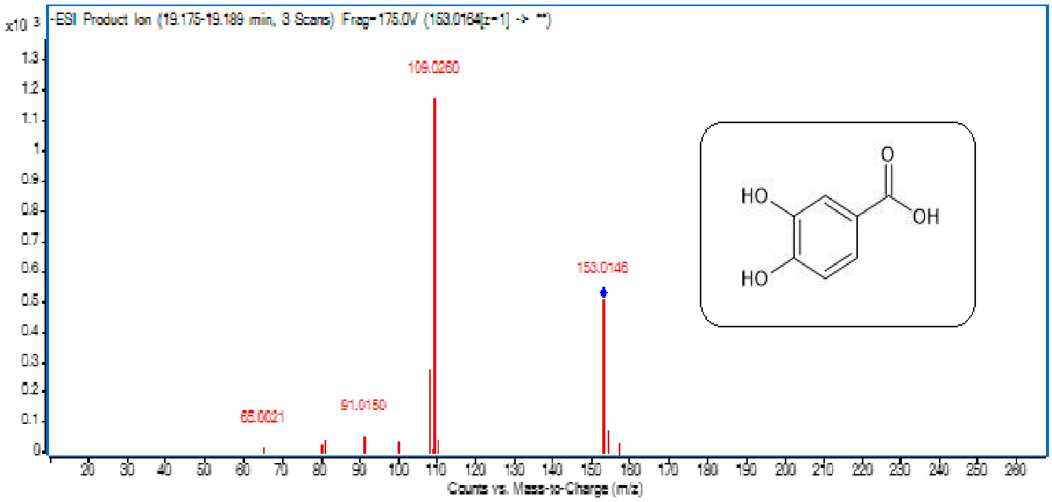

Figure S2. MS/MS spectra of (a) gallic acid and (b) protocatechuic acid.

S2.3.3. Alpha and beta-diversity

Alpha diversity metrics (Observed ASVs, Simpson's evenness, and Shannon Diversity) (Figure S2) identified apparent biological differences between the three different groups (MPP, YA, and YC) after 72 hours of fermentation. In both yoghurt samples (YA and YC), there was an apparent reduction in the total number of ASVs between 24 hours and 72 hours of fermentation. There was however a decrease in both diversity and evenness in the YA

samples, but an increase in these metrics in the YC samples. The low diversity in YC samples at 24 hours are reflected in the relative abundance, which highlight that the population is dominated by Streptococcus spp., which is altered at the 72-hour mark wherein the YC samples have a higher diversity. (Le Roy et al., 2022) reported transient increase of yoghurt contained species (i.e., *Streptococcus thermophillus*) following ingestion of yoghurts. Interestingly the samples that only contain MPP had the highest diversity at both 24 and 72 hours.

The broader population structures, measured through beta-diversity highlight that the YC samples, both quantitatively (weighted UniFrac) and qualitatively (unweighted UniFrac) (Figure S3), have substantial dissimilarity compared to the MPP and YA samples at 24 hours. It suggests that the addition of probiotics results in an alteration of the microbiota population structure compared to samples without these additions. After 72 hours the dissimilarity between YA and YC microbiota appear to be reduced.

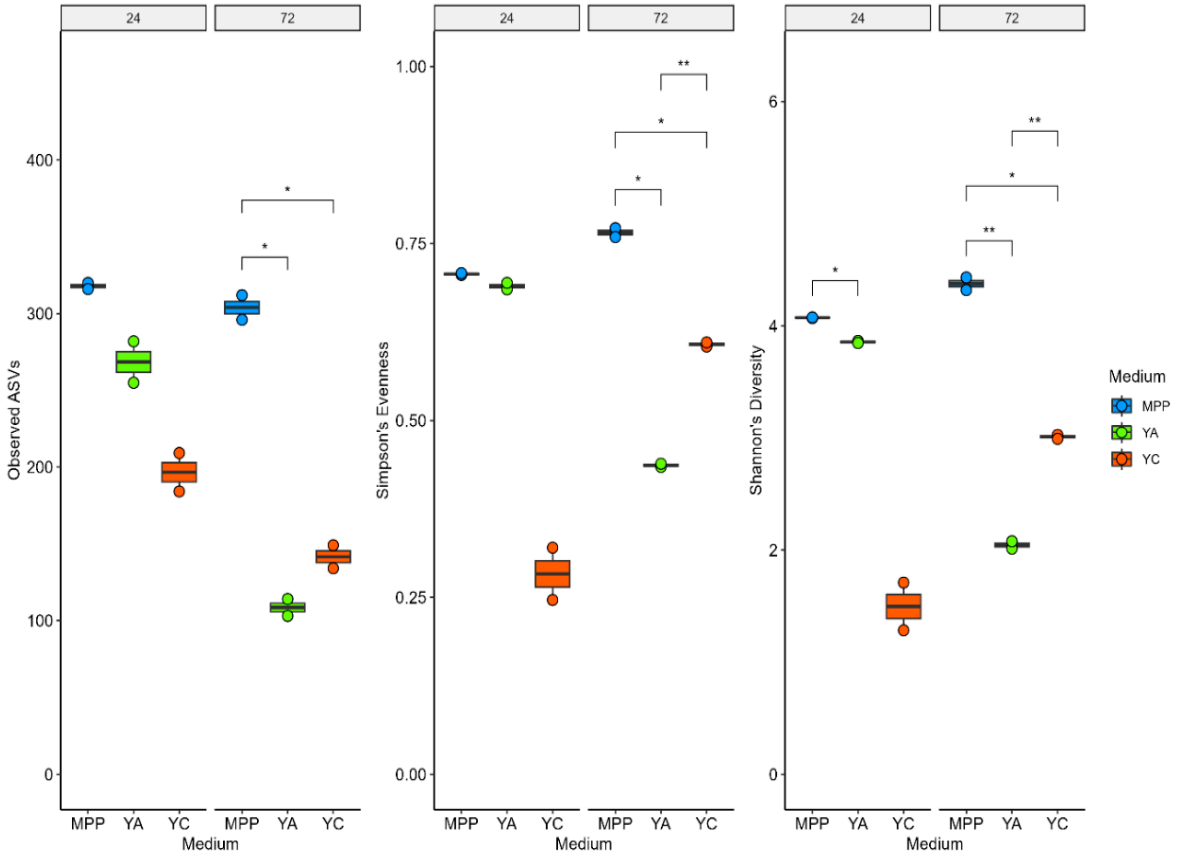

Figure S3. Estimated alpha diversity of microbiome samples (Observed ASVs, Simpson's Evenness, and Shannon Entropy) at 24 and 72 h. Significance between groups as determined by a student's t-test with adjustment for multiple comparisons denoted by * ($p < 0.05$) and ** ($p < 0.01$).

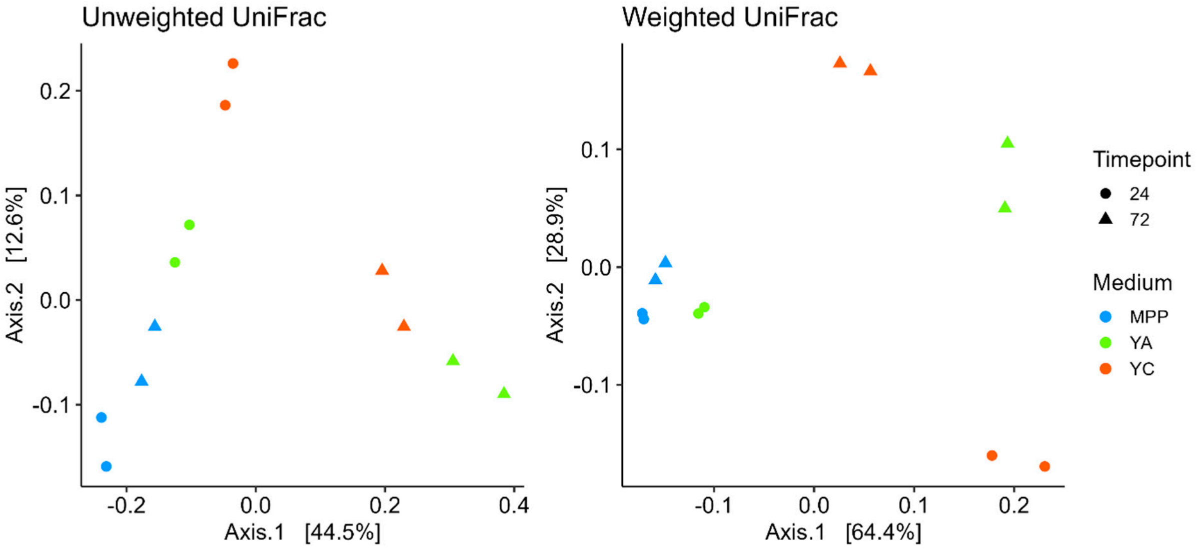

Figure S4. Beta-diversity of microbial populations from principal coordinate analysis of Unweighted and Weighted UniFrac ordination. All samples were rarefied to 46,338 reads for ordination analysis.
